# RhoC Impacts the Metastatic Potential and Abundance of Breast Cancer Stem Cells

**DOI:** 10.1371/journal.pone.0040979

**Published:** 2012-07-23

**Authors:** Devin T. Rosenthal, Jie Zhang, Liwei Bao, Lian Zhu, Zhifen Wu, Kathy Toy, Celina G. Kleer, Sofia D. Merajver

**Affiliations:** 1 Division of Hematology and Oncology, Department of Internal Medicine, University of Michigan, Ann Arbor, Michigan, United States of America; 2 Cellular and Molecular Biology Program, University of Michigan, Ann Arbor, Michigan, United States of America; 3 Department of Pathology, University of Michigan, Ann Arbor, Michigan, United States of America; 4 University of Michigan Comprehensive Cancer Center, University of Michigan, Ann Arbor, Michigan, United States of America; The Beatson Institute for Cancer Research, United Kingdom

## Abstract

Cancer stem cells (CSCs) have been shown to promote tumorigenesis of many tumor types, including breast, although their relevance to cancer metastasis remains unclear. While subpopulations of CSCs required for metastasis have been identified, to date there are no known molecular regulators of breast CSC (BCSC) metastasis. Here we identify RhoC GTPase as an important regulator of BCSC metastasis, and present evidence suggesting that RhoC also modulates the frequency of BCSCs within a population. Using an orthotopic xenograft model of spontaneous metastasis we discover that RhoC is both necessary and sufficient to promote SUM149 and MCF-10A BCSC metastasis–often independent from primary tumor formation–and can even induce metastasis of non-BCSCs within these cell lines. The relationship between RhoC and BCSCs persists in breast cancer patients, as expression of RhoC and the BCSC marker ALDH1 are highly correlated in clinical specimens. These results suggest new avenues to combating the deadliest cells driving the most lethal stage of breast cancer progression.

## Introduction

In the majority of cancers it is not the primary tumor that is lethal to the patient; the actual lethality arises from cancer cell metastasis to vital organs. Recent work has uncovered emerging roles for cancer stem cells (CSCs) in cancer metastasis. Initial links between CSCs and metastasis were circumstantial, including an invasiveness gene signature in breast CSCs (BCSCs) that predicted shorter metastasis-free survival [1] and an association between BCSCs and the metastasis-associated epithelial-to-mesenchymal transition [2].

Recent studies suggested stronger causative links between BCSCs and metastasis. BCSCs have been found to be enriched in spontaneous breast cancer xenograft metastases [3], and CSC subpopulations that selectively enable pancreatic and colon cancer metastasis have been identified [4], [5]. While evidence for CSCs acting in metastasis exists and markers identifying metastatic CSC populations are emerging, a functional molecular link between BCSCs and metastasis has not been identified. Here we discover that RhoC GTPase can promote BCSC metastasis and can initiate metastasis independent of primary tumor formation.

RhoC is a member of the Rho family of GTPases and functions in coordinating cell motility and actomyosin contractility [6], [7]. RhoC promotes metastasis of many cancers [8], [9], [10], [11]. Moreover, RhoC knockout selectively inhibits metastasis–independent from primary tumor formation–in a transgenic breast cancer model [12]. Clinically, RhoC expression increases with breast cancer progression, and high RhoC expression is significantly associated with decreased patient survival [13].

The metastatic influence of RhoC is exemplified by inflammatory breast cancer (IBC). IBC is the most lethal form of breast cancer and is metastatic from its inception. RhoC is overexpressed in 90% of IBC cases [14]; furthermore, RhoC overexpression partially recapitulates the IBC phenotype *in vitro* [8]. BCSCs, defined by the BCSC and hematopoietic stem cell marker aldehyde dehydrogenase (ALDH) [15], [16], and RhoC have been shown to independently function in IBC metastasis and are separately associated with poor clinical outcome [8], [13], [17]. Due to the strong associations between RhoC, BCSCs, and IBC metastasis, we hypothesized that RhoC functionally contributes to BCSC pathogenesis.

Here we reveal that RhoC can function in BCSC metastasis. Inhibiting RhoC in the highly metastatic, IBC-derived SUM149 cell line revealed that RhoC is necessary for SUM149 BCSC metastasis. Conversely, overexpressing RhoC alone was sufficient to enable BCSC metastasis from the non-tumorigenic, non-metastatic MCF-10A cell line. Surprisingly, RhoC often promoted spontaneous metastasis independent from primary tumor formation even within the non-BCSC population, suggesting that RhoC can act independent of BCSC status. RhoC also influences BCSC population size in the cell lines studied, as the abundance of BCSCs varied concurrent with changes in RhoC expression. Clinically, expression of RhoC and the BCSC marker ALDH1 strongly correlate in patient breast cancer specimens. To the best of our knowledge RhoC is therefore the first putative molecular promoter of BCSC metastasis–one which holds therapeutic promise for the most lethal form of breast cancer.

## Results

### RhoC Expression is Enriched in ALDH (+) BCSCs

**Figure 1:**
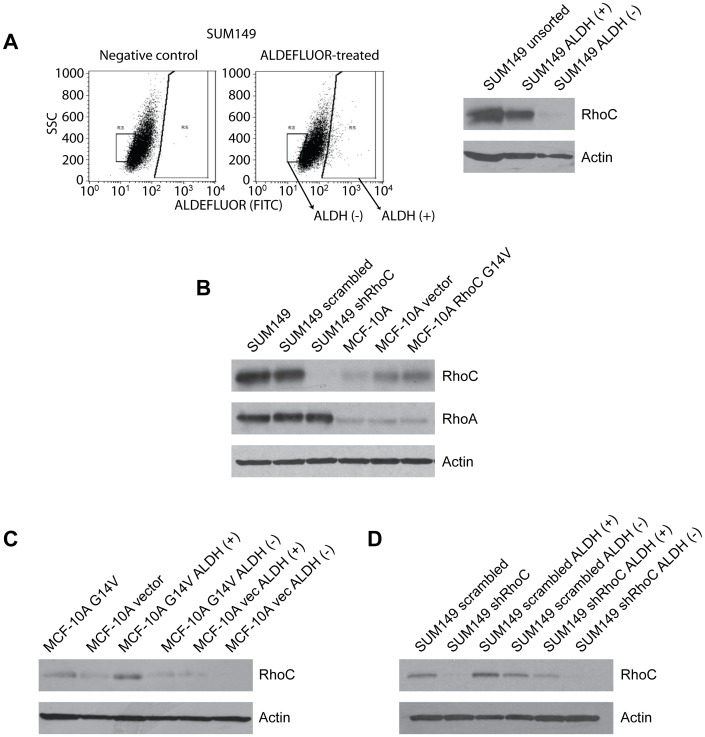
RhoC expression is intimately linked to the ALDH (+) breast cancer stem cell population in SUM149 and MCF-10A G14V cells. (**A**) (left) ALDH (+) and (−) cells were isolated by FACS of ALDEFLUOR-treated SUM149 cells. (right) RhoC expression is linked to BCSC status in SUM149 IBC cells. (**B**) Generation of RhoC knockdown SUM149 cells and constitutively active RhoC (RhoC G14V)-overexpressing MCF-10A cells. Importantly, modulating RhoC expression did not affect expression of the close homolog RhoA. (**C–D**) Interestingly, even when RhoC is exogenously expressed or inhibited in MCF-10A (**C**) or SUM149 cells (**D**), RhoC expression still segregates to the ALDH (+) population.

To address whether RhoC functions in BCSC pathogenesis, we first asked whether RhoC expression was associated with BCSCs. Using the highly aggressive, RhoC-overexpressing SUM149 cell line we discovered that, after sorting for ALDH activity using the ALDEFLUOR assay [16] (Figure 1A, left), RhoC expression was primarily confined to the ALDH (+) SUM149 BCSC population (Figure 1A, right) and its expression is homogenous within the ALDH (+) population (data not shown), suggesting that RhoC is associated with BCSCs.

To determine whether RhoC functions in BCSC aggressiveness, we generated genetically modified cell lines with either inhibited RhoC in SUM149 cells (“SUM149 shRhoC”) or overexpressed constitutively active RhoC [18], [19] in the non-tumorigenic mammary epithelial cell line MCF-10A (“MCF-10A G14V”) (Figure 1B). Importantly, neither modification affected expression of the close RhoC homolog RhoA (Figure 1B).

Interestingly, when we sorted the modified cell lines and observed RhoC expression as in Figure 1A, we found that RhoC was still enriched in the ALDH (+) population even within the genetically modified cells (Figure 1C–D). This was surprising, given that these cells were either forcibly overexpressing (Figure 1C) or knocking down (Figure 1D) RhoC. The fact that this dichotomy in RhoC expression persisted after genetic modification strengthened the case for an association between BCSCs and RhoC.

### Modifying RhoC Expression Alters the *in vitro* Metastatic Properties of SUM149 and MCF-10A G14V BCSCs

Upon observing a strong association between RhoC expression and activity of the BCSC marker ALDH, we asked whether this relationship was functional in BCSC behavior. The acquisition of motility by otherwise stationary cells is an indicator of cancer progression and a process regulated across many cell lineages and cancers by RhoC [8], [9], [20]. Because CSCs have been linked to metastasis, albeit indirectly, we investigated RhoC influence on BCSC motility using time lapse microscopy.

**Figure 2:**
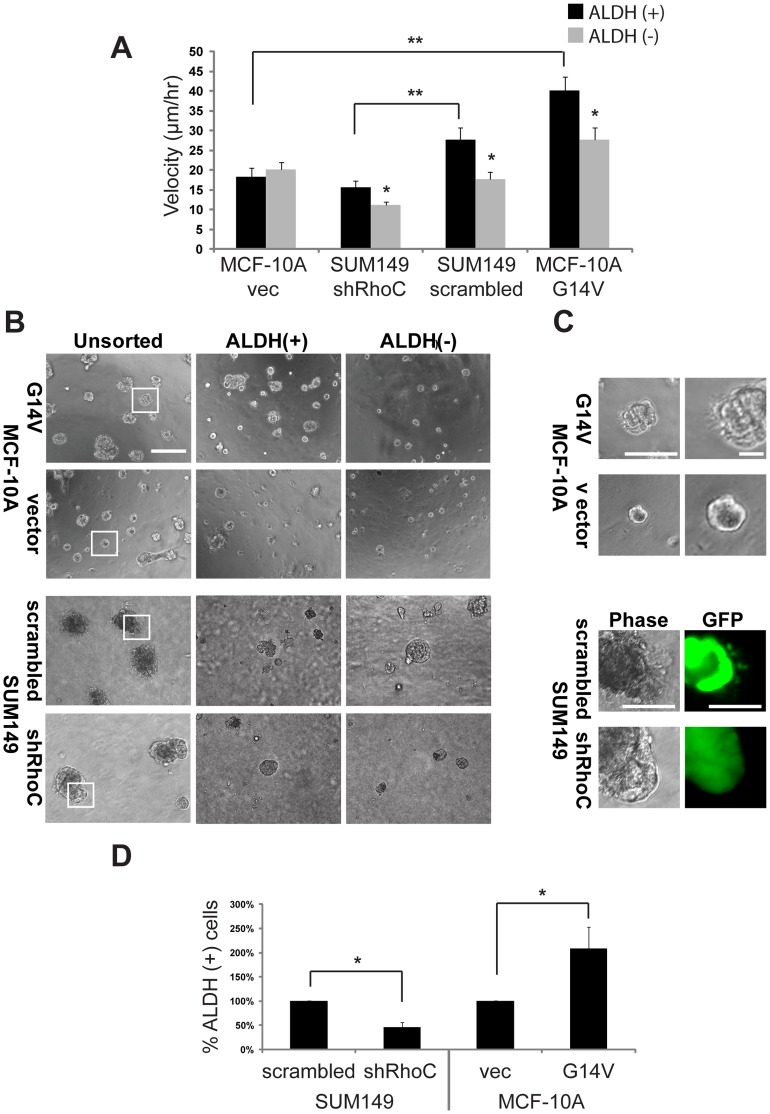
RhoC dictates the behavior and abundance of SUM149 and MCF-10A BCSCs. (**A**) RhoC expression determines cell speed, even within the ALDH (+) population. Comparing the ALDH (+) populations of each cell line, modulating RhoC expression causes corresponding changes in cell speed (i.e. decreased cell speed in ALDH (+) SUM149 shRhoC compared to SUM149 scrambled). Comparing between ALDH populations within a cell line, cell speed is decreased in the ALDH (2) population concurrent with RhoC expression (Figure 1C–D) (*p,0.05; **p,0.01; MCF-10A vec n  = 93(+), 128(2); MCF10A G14V n  = 85(+), 92(2); SUM149 scrambled n  = 66(+), 52(2); SUM149 shRhoC n  = 67(+), 76(2)). (**B**) Cell growth in 3D Matrigel culture reflects RhoC expression. Cells with high RhoC expression (SUM149 scrambled and MCF-10A G14V) exhibit aggressive, invasive growth in 3D culture, whereas cells with low RhoC expression (SUM149 shRhoC and MCF-10A vec) do not invade into the surrounding matrix (see “unsorted”). When sorted for ALDH, this invasive outgrowth is restricted to the ALDH (+) fraction of the high RhoC-expressing cell lines, suggesting that ALDH (+) BCSC aggressiveness in these cell lines is reliant on RhoC (scale  = 100 mm). (**C**) Enlarged representative images of the indicated MCF- 10A cells (top) and SUM149 cells (bottom) from (B) illustrating the invasive outgrowths in cell populations expressing RhoC. GFP that is ubiquitously expressed from the pGIPz shRNA plasmid clearly shows invasive cellular outgrowths in SUM149 scrambled, but not shRhoC, cells (bottom) (scale  =  50 mm (MCF-10A G14V, left, and all SUM149 images) and 12.5 mm (MCF-10A G14V, right)). (**D**) In addition to modifying the behavior of ALDH (+) cells, RhoC expression also alters the abundance of ALDH (+) BCSCs within a cell line. The relative number of ALDH (+) cells within the SUM149 population decreases by over 50% in SUM149 shRhoC compared to SUM149 scrambled cells and is doubled in MCF-10A G14V compared to MCF-10A vec cells. All error bars represent the standard error of the mean (SEM).

Modulating RhoC expression significantly impacted cell velocity even within the ALDH (+) BCSC population in each cell line (Figure 2A). Inhibiting RhoC in highly motile SUM149 cells significantly reduced, while overexpressing RhoC in slow-moving MCF-10A cells significantly increased, cell speed. Interestingly, we also observed significant cell speed differences between ALDH (+) and (−) cells within each cell line, again paralleling RhoC expression. Decreased RhoC in SUM149 cells (either by shRNA or within the ALDH (−) population) reduced cell motility to levels comparable to MCF-10A control cells (“MCF-10A vec”). Even in the highly motile MCF-10A G14V cell line, ALDH (−) cells (with lower RhoC G14V expression than ALDH (+) cells (Figure 1C)) were slower than ALDH (+) cells. The only exception to this dichotomy was MCF0-10A vec cells, which is not entirely unexpected as this cell line is non-tumorigenic, slow-moving, and has low RhoC expression (Figure 1B–C).

3D cell culture is frequently used to observe physiologically-relevant developmental, tumorigenic, and metastatic mammary epithelial/breast cancer cell behaviors *in vitro* [21], [22]. We employed this technique to characterize the RhoC-modified and ALDH-sorted cells. As has previously been observed [21], [22], unsorted MCF-10A vec cells formed small, well-defined acinar-like structures (Figure 2B, column 1, row 2). By contrast, SUM149 scrambled cells grew as large, disorganized clusters that formed invasive protrusions into the surrounding matrix (Figure 2B, column 1, row 3)–growth characteristic of tumorigenic, metastatic breast cancer cells [22], [23].

Modifying RhoC expression significantly impacted the *in vitro* metastatic phenotype of both unsorted cell lines. MCF-10A G14V acinar-like structures were more disorganized and slightly larger than the MCF-10A vec acinar-like, with cells invading out from the central mass, similar to SUM149 scrambled cells (Figure 2B, column 1, row 1, and Figure 2C, top). Conversely, SUM149 shRhoC cells formed structures of comparable size to SUM149 scrambled cells; however, these structures had well-defined borders, showing minimal evidence of cell invasion into the matrix (Figure 2B, column 1, row 4, and Figure 2C, bottom).

The effects of ALDH sorting once again mirrored RhoC expression within each cell line. ALDH (+) cells (Figure 2B, second column) grew similarly to the unsorted population (Figure 2B, column 1), whereas ALDH (−) cells appeared both non-tumorigenic and non-invasive in all cell lines (Figure 2B, column 3). Of note, although some SUM149 scrambled ALDH (−) colonies formed large acinar-like structures, these structures had well-defined borders with no signs of invasive behavior (Figure 2B, column 3, row 3). Taken together, these data support a role for RhoC in mediating metastatic behaviors of SUM149 and MCF-10A G14V ALDH (+) BCSCs.

### Modulating RhoC Expression Causes Concurrent Changes in the Abundance of SUM149 and MCF-10A ALDH (+) Cells

While ALDH-sorting we made a surprising observation: there appeared to be a RhoC-dependent change in the abundance of ALDH (+) cells in each cell line. To further investigate, we compared the relative abundance of ALDH (+) cells in each control cell line (SUM149 scrambled or MCF-10A vec) to the corresponding RhoC-modified cell line (SUM149 shRhoC or MCF-10A G14V). Surprisingly, we observed almost identical reciprocal changes: a two-fold decrease in the relative number of ALDH (+) cells in the SUM149 shRhoC cell line compared to scrambled, and a two-fold increase in the relative number of ALDH (+) cells in the MCF-10A G14V cell line compared to vector (Figure 2D). Together, these data suggest that RhoC expression may affect the abundance of BCSCs within a population.

### RhoC Expression Dictates SUM149 and MCF-10A BCSC Metastasis

Based on our *in vitro* observations, we asked whether RhoC affects SUM149 and MCF-10A BCSC metastasis *in vivo*. To address this question, we orthotopically xenografted NOD/SCID mice with either ALDH-sorted SUM149 scrambled/shRhoC cells or MCF-10A vec/G14V cells (see Materials and Methods) and observed the incidence of tumorigenesis and metastasis.

Knocking down RhoC in SUM149 cells significantly decreased tumor incidence in the ALDH (+) population (see Table 1). 5 of 9 (55.6%) ALDH (+) SUM149 scrambled control mice developed tumors, whereas 0 of 8 mice injected with ALDH (+) SUM149 shRhoC cells formed tumors (p = 0.029). Only one ALDH (+) MCF-10A G14V mouse developed a tumor compared to zero ALDH (+) MCF-10A vec (not significant) (Table 1). At the limiting cell numbers used none of the mice injected with ALDH (−) cells formed tumors.

**Table 1:** Analysis of xenografted mice.

Cell Line	ALDH	Total Mice	Tumors		Lung Metastases		p-value for ALDH status (mets)	p-value between ALDH+ based on RhoC status (mets)
MCF-10A vector	+	8	0		0		**n.s.**	
	−	7	0		0			
MCF-10A G14V	+	10	**1**	**(10%)**	**9**	**(90%)**	**0.035**	**0.0002**
	−	6	0		2	(33.33%)		
SUM149 scrambled	+	9	**5**	**(55.56%)**	**6**	**(66.67%)**	**0.0045**	
	−	9	0		0			
SUM149 shRhoC	+	8	0		**1**	**(12.50%)**	**n.s.**	**0.0364**
	−	8	0		0			

**Figure 3:**
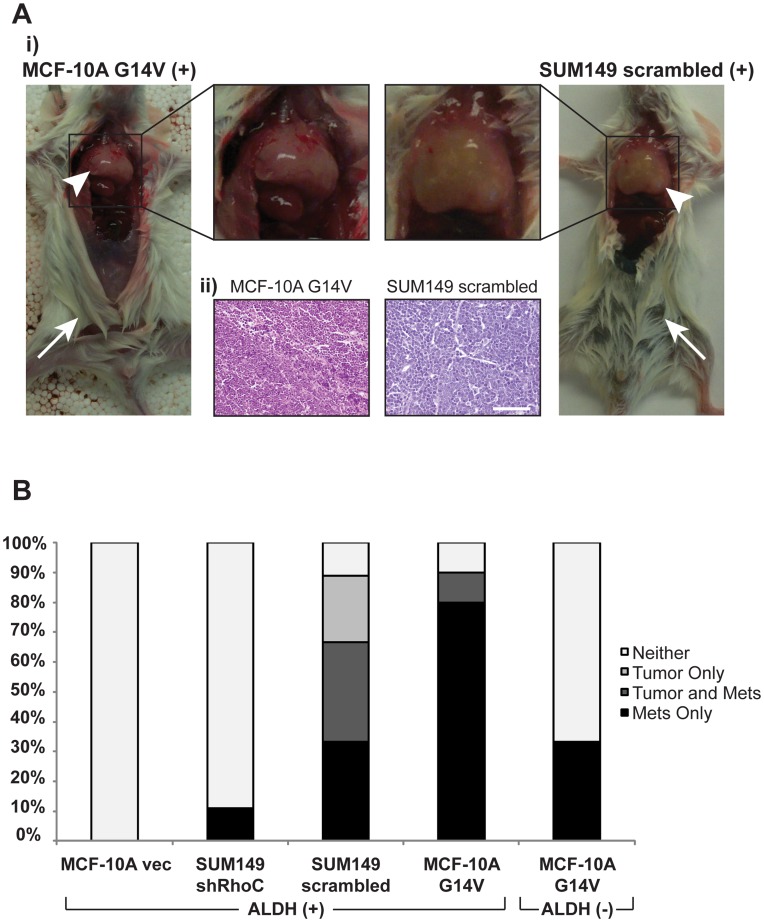
RhoC expression determines metastasis of SUM149 and MCF-10A ALDH (+) BCSCs. (**A**) NOD/SCID mice orthotopically xenografted with only 50 SUM149 or 5000 MCF-10A G14V cells form large lung metastases, often independent of primary tumor formation. **i**) Arrows indicate injection sites lacking primary tumors, arrowheads denote lung metastases. **ii**) H&E-staining shows that lung metastases from both MCF-10A G14V and SUM149 scrambled cells are poorly-differentiated invasive carcinomas (scale  = 50 µm) (**B**) Quantitative analysis of xenografted mice establishes RhoC as both necessary and sufficient for SUM149 and MCF-10A G14V ALDH (+) BCSC lung metastasis (also see Table 1). MCF-10A cells overexpressing RhoC G14V metastasize independent of primary tumor formation. Note that even ALDH (−) MCF-10A G14V, which have reduced but not completely eliminated constitutively active RhoC expression, do not form primary tumors but can still metastasize, albeit less frequently, than ALDH (+) MCF-10A G14V. Importantly, the overall incidence of cancer drops from greater than 85% in ALDH (+) SUM149 scrambled and MCF-10A G14V mice to less than 13% in ALDH (+) SUM149 shRhoC and MCF-10A vec mice, demonstrating the essential role RhoC plays in ALDH (+) BCSC aggressiveness of these cell lines *in vivo*.

Surprisingly, we discovered large metastatic tumors completely filling the pleural cavity in many of the mice injected with ALDH (+) SUM149 scrambled and MCF-10A G14V cells (Figure 3Ai). We also observed one instance in the ALDH (+) SUM149 shRhoC cohort and two instances in the ALDH (−) MCF-10A G14V cohort (Figure 3B and Table 1). Histological examination revealed these tumors to be poorly differentiated carcinomas, with remarkably similar appearance between the MCF-10A G14V and SUM149 scrambled metastases (Figure 3Aii). In all, 66.67% of ALDH (+) SUM149 scrambled- and 90% of ALDH (+) MCF-10A G14V-injected mice presented with metastases, compared to only 12.5% of ALDH (+) SUM149 shRhoC- and 33.33% of ALDH (−) MCF-10A G14V-injected mice (Table 1).

Even more surprising was the propensity for metastasis in mice that did not form primary tumors (Figure 3A–B). 35% of ALDH (+) SUM149 scrambled-injected, and a remarkable 80% of ALDH (+) MCF-10A G14V-injected, mice had metastases independent of primary tumor formation (Figure 3B). Additionally, all of the ALDH (+) SUM149 shRhoC and ALDH (−) MCF-10A G14V mice presenting with metastases also lacked primary tumors (Figure 3B). Pathological examination of the injection site confirmed that no injected cells were present in the mammary gland.

One central tenant of the CSC hypothesis is that CSCs can self-renew and generate heterogeneity within a tumor whereas non-CSCs cannot [24]. Based on this assumption, we hypothesized that the dichotomy in RhoC expression between the SUM149 ALDH (+) and (−) populations would be maintained *in vivo*, such that RhoC-low, ALDH (−) SUM149 cells would not be able to generate RhoC-high, ALDH(+) cells, and thus ALDH (−) SUM149 tumors would retain low RhoC expression. Conversely, RhoC-high, ALDH(+) tumors would retain the high RhoC expression characteristic of the unsorted SUM149 cell line. We used the SUM149 scrambled cell line to assay RhoC expression *in vivo*, as it is the only cell line in this study that expresses high levels of endogenous RhoC (Figure 1B).

**Figure 4:**
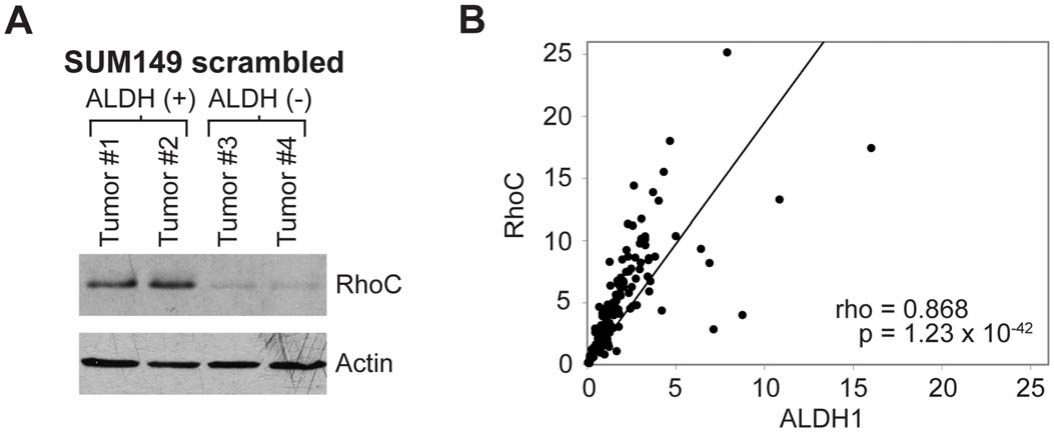
RhoC is tightly associated with the BCSC marker ALDH1 in both xenograft and patient tumors. (**A**) Injecting mice with 5000 SUM149 scrambled cells–at which point both ALDH (+) and ALDH (−) cells form tumors–reveals that ALDH (−) tumors maintain reduced RhoC levels *in vivo*. This provides mechanistic evidence for the inability of ALDH (−) cells to metastasize (their inability to re-express RhoC), and speaks to the inability of ALDH (−) cells to reconstitute tumor heterogeneity and thus restore tumor RhoC expression through the expansion of RhoC-expressing ALDH (+) cells. (**B**) RhoC and ALDH1 expression are highly correlated in clinical breast cancer samples (Spearman’s rho  = 0.868, p = 1.23×10^−42^, df = 134).

Since none of the mice injected with 50 ALDH (−) SUM149 scrambled cells formed tumors we increased the injection to 5000 cells, at which point the ALDH (−) population also formed tumors. After allowing tumors to develop, we euthanized the mice, extracted protein from the tumors, and assayed RhoC expression. The inverse relationship between RhoC expression and ALDH activity persisted *in vivo*; ALDH (+) SUM149 scrambled tumors maintained RhoC expression during tumor growth, whereas ALDH (−) SUM149 scrambled tumors did not regain RhoC expression (Figure 4A). These data support the link between RhoC and SUM149 BCSCs, as ALDH (−) SUM149 scrambled cells failed to restore tumor heterogeneity and re-express RhoC after expansion *in vivo*. Along with our previous findings illustrating the influence of RhoC on SUM149 and MCF-10A BCSC abundance (Figure 2C), these data further support the notion that RhoC expression may be intimately linked to the BCSC phenotype.

### Expression of RhoC and the BCSC Marker ALDH1 are Highly Correlated in Clinical Breast Cancer Samples

Expression of ALDH1 protein has been shown to be a reliable marker for BCSCs in paraffin-embedded tissue [16]. To extend our findings on the relationship between RhoC and BCSCs we used AQUA of immunofluorescence signals for RhoC and ALDH1 in cytokeratin-positive cells from a breast cancer tissue microarray. Expression of RhoC and ALDH1 were strongly positively correlated in the 136 samples analyzed (Figure 4B), indicating a tight association between RhoC and a BCSC marker in breast cancer patients. Taken together with our *in vitro* and *in vivo* data, these findings further support an association between RhoC and BCSCs.

## Discussion

CSCs have been shown to promote tumorigenesis in numerous cancer types [25], [26], [27], and recent work has begun to define a role for CSCs in cancer metastasis as well [3], [4], [5], [17]. Despite the established therapeutic importance of targeting metastasis and a growing understanding of the role CSCs play in metastasis, to date no functional molecular regulators have been identified that promote aggressive, metastatic BCSC behavior. Here we identify RhoC as a potential promoter; one that is both necessary and sufficient for SUM149 and MCF-10A BCSC metastasis.

By approaching RhoC expression from two distinct angles–its necessity for metastasis of a breast cancer cell line (SUM149), and its sufficiency to induce metastasis of a non-metastatic mammary epithelial cell line (MCF-10A)–we were able to clearly elucidate a putative role of RhoC in BCSC metastasis. It is important to note that we used an orthotopic xenograft system rather than an intracardiac or tail vein injection assay to measure metastasis. As emphasized in a recent publication [3], the orthotopic xenograft model of spontaneous breast cancer metastasis is a more physiologically relevant model of breast cancer metastasis, which more accurately recapitulates the microenvironmental obstacles metastatic cells encounter in human patients.

We have shown that RhoC expression and BCSC marker expression are intimately linked in multiple ways (Figures 1C–D, 2C). Taken together, these data reveal a close association between RhoC and SUM149/MCF-10A BCSCs; one in which RhoC expression determines SUM149/MCF-10A BCSC metastatic potential and may also contribute to BCSC frequency within the cell lines. Furthermore, we demonstrated that this relationship between RhoC and the BCSC marker ALDH1 persists in a heterogeneous patient population, suggesting that RhoC may indeed be linked to BCSCs beyond the SUM149 and MCF-10A cell lines. This evidence supports the theory that a larger BCSC population–and thus higher RhoC expression–may confer a worse prognosis [13], [28].

We were surprised to find that a significant number of mice developed pleural metastases independent of primary tumor formation (Figure 3 and Table 1). We initially hypothesized that these metastases may have resulted from improper injection into the mammary gland; however, identical metastases were observed in both tumor-bearing and tumor-free mice, which strongly suggests that the metastases in tumor-free mice did not result from improper injection.

As an alternative explanation, we propose that high RhoC expression–either as a consequence of inherent tumor cell biology (SUM149) or genetic modification (MCF-10A G14V), and amplified by ALDH (+) status–may, in some cases, cause a sublimation of cell behavior from non-tumorigenic directly to metastatic. Such a phenomenon is observed clinically and is defined as cancer of unknown primary site, or CUP [29]. Given that RhoC primarily mediates motility, invasion, and angiogenesis [8], [14]–all metastasis-associated properties–it stands to reason that RhoC may be capable of driving metastatic progression independent from primary tumor formation.

Interestingly, several mice injected with non-CSC ALDH (−) MCF-10A G14V still developed lung metastases (Table 1). That these cells were metastatic further supports RhoC sufficiency to induce metastasis. Although ALDH (−) MCF-10A G14V cells had lower RhoC expression, RhoC was not completely eliminated (Figure 1C)–as is to be expected from a cell line forcibly overexpressing a transgene. Furthermore, the residual RhoC is predominately constitutively active RhoC, thus amplifying the effects of even low expression levels. Therefore, the fact that several ALDH (−) MCF-10A G14V mice developed metastases is not surprising and supports the hypothesis that RhoC may be able to promote metastasis independent of BCSC status–although, under normal conditions, RhoC expression remains closely associated with the BCSC population.

In agreement with this assertion, we also observed metastasis in one mouse injected with ALDH (+) SUM149 shRhoC cells. As we previously observed, RhoC is preferentially expressed by the ALDH (+) SUM149 population (Figures 1A, C–D), and although we achieved significant RhoC knockdown, RhoC expression was not completely eliminated from SUM149 shRhoC cells (Figure 1D). Accordingly, the remaining RhoC was primarily confined to the ALDH (+) population (Figure 1D). Again, though correlative, this evidence supports the hypothesis that RhoC expression is both necessary and sufficient for SUM149 BCSC metastasis and is intimately linked to the BCSC population.

These observations raise important questions about the true meaning of CSC identity. Labeling a cell a CSC may indicate that it has a specific collection of features (i.e. unlimited replication potential, increased metastatic potential, and others), but ultimately these features are a product of the genetics of the CSC. Extending from this assumption, one can reason that targeting the specific molecular cogs driving the CSC machinery–rather than focusing on incidental markers that delineate CSCs–may have therapeutic potential. Eliminating CSCs may ultimately be necessary to cure certain cancers, but disrupting the molecular CSC machinery may be able to manage the CSC population in the interim.

The work presented here provides strong rationale for therapeutically targeting RhoC. RhoC was previously shown to be essential for metastasis [12] and is overexpressed in many different cancers [9], [10], [11]–in particular IBC, which presently lacks effective therapies [14]–yet this is the first work relating RhoC to BCSCs. To this end, our lab has designed a small molecule RhoC inhibitor that has shown good *in vitro* and *in vivo* efficacy with no apparent toxicity (unpublished data). As therapies targeting CSCs emerge [30], it will be important to address which CSC population is being targeted–the tumorigenic or the metastatic population–in order to effectively combat the disease.

## Materials and Methods

### Reagents

The constitutively active RhoC expression plasmid (RhoC G14V in pcDNA3.1) was purchased from the Missouri S&T cDNA Resource Center (www.cdna.org). RhoC shRNA and the scrambled control plasmid came from Origene and were obtained through the University of Michigan shRNA core facility. The shRNA sequence used was 5′-CCGTCCCTACTGTCTTTGAGAA-3′. shRNA was expressed off of either the pSM2c (to allow for ALDEFLUOR sorting) or pGIPZ (to allow for fluorescent imaging) plasmids.

### Electroporation

Cell lines stably expressing either RhoC shRNA, RhoC G14V, or the respective control plasmids were generated by nucleofection with the target plasmid using an Amaxa Nucleofector (Lonza). Nucleofected cells were selected for and maintained using the appropriate antibiotic (1 µg/ml puromycin for shRNAs; 350 ng/ml neomycin for overexpression plasmids) and grown as pooled populations of nucleofected cells.

### 3D Cell Culture

Cell lines were cultured as previously described by Lee et al. [22] using the “on-top” method. Four-well chamber slides (Lab-Tek) were coated with growth factor-reduced Matrigel (BD Biosciences). Cells were then plated at a density of 2.1×10^4^ cells/cm^2^ on top of the Matrigel coating. Cell media containing 4% Matrigel was then added and the cells were cultured for 6–10 days before imaging.

### Time-Lapse Microscopy

DIC time-lapse videos were captured at 37°C using a Deltavision RT Live Cell Imaging System and acquired using SoftWoRx 3.5.1 software. Cells were cultured in 2D on collagen-coated chamber slides, and images were taken every 10 minutes for 18 hours. The velocities of individual cells were tracked manually using the MTrackJ plugin (http://www.imagescience.org/meijering/software/mtrackj/) for ImageJ [31].

### ALDEFLUOR Assay

The ALDEFLUOR assay was performed according to the manufacturer’s instructions; see also [16]. Briefly, ALDEFLUOR-treated cells quenched with DEAB were used to set the ALDEFLUOR-positive FACS gate, which we defined as a gate containing less than 0.01% of DEAB-treated cells. Cells treated with ALDEFLUOR alone were then sorted by FACS and used for downstream experiments.

### Orthotopic Xenografts and Metastasis Analysis

All mouse work was approved by the University of Michigan’s University Committee on Use and Care of Animals (protocol #09685). After sorting into ALDH (+) and (−) populations, cells were diluted 1∶1 with Matrigel (BD Biosciences). NOD/SCID mice were anesthetized, the mammary fat pad was exposed, and the mice were injected with either 50 (SUM149 and variants) or 5000 (MCF-10A and variants) cells directly into the fourth mammary gland. Tumors were monitored weekly and mice were euthanized once tumor volume approached 2 cm^3^ or mice showed signs of morbidity. Mice that did not develop tumors or show signs of morbidity were euthanized at the latest time point for their comparative cohort. Lungs were analyzed at the time of euthanization for macroscopic metastases. Tumors and lungs were then resected, fixed in 10% formalin, paraffin embedded, and stained with hematoxylin and eosin.

### In Situ Detection and Quantification of Protein Expression

#### Tumors and patients

Fresh and formalin-fixed, paraffin-embedded breast cancer tissue blocks were obtained from the Department of Pathology files at the University of Michigan Medical Center. Ethics approval was obtained from the Institutional Review Board at the University of Michigan. Written consent was obtained from all patients, and diagnoses were confirmed by morphology. After pathological review, a tissue microarray was constructed from the most representative area using the methodology of Nocito et al. [32].

#### Immunohistochemical staining and AQUA analysis

Triple immunofluorescence staining was performed as previously described [33] and the AQUA system (HistoRx, New Haven, Connecticut) was used for automated image acquisition and analysis. A detailed staining and imaging procedure can be found in Methods S1.

### Statistical Analysis

All p-values were calculated by Student’s two-tailed t-test unless otherwise noted. Expression levels of ALDH1 and RhoC in TMA samples were compared using Spearman’s rank coefficient.

## Supporting Information

Methods S1 **Supplemental materials and methods.**
(DOC)

## References

[1] Liu R, Wang X, Chen GY, Dalerba P, Gurney A (2007). The prognostic role of a gene signature from tumorigenic breast-cancer cells.. N Engl J Med.

[2] Mani SA, Guo W, Liao MJ, Eaton EN, Ayyanan A (2008). The epithelial-mesenchymal transition generates cells with properties of stem cells.. Cell.

[3] Liu H, Patel MR, Prescher JA, Patsialou A, Qian D (2010). Cancer stem cells from human breast tumors are involved in spontaneous metastases in orthotopic mouse models.. Proc Natl Acad Sci U S A.

[4] Hermann PC, Huber SL, Herrler T, Aicher A, Ellwart JW (2007). Distinct populations of cancer stem cells determine tumor growth and metastatic activity in human pancreatic cancer.. Cell Stem Cell.

[5] Pang R, Law WL, Chu AC, Poon JT, Lam CS (2010). A subpopulation of CD26+ cancer stem cells with metastatic capacity in human colorectal cancer.. Cell Stem Cell.

[6] Rosenthal DT, Brenner JC, Merajver SD, van Golen KL (2010). Rho Proteins in Cancer..

[7] Wheeler AP, Ridley AJ (2004). Why three Rho proteins? RhoA, RhoB, RhoC, and cell motility.. Exp Cell Res.

[8] van Golen KL, Wu ZF, Qiao XT, Bao LW, Merajver SD (2000). RhoC GTPase, a novel transforming oncogene for human mammary epithelial cells that partially recapitulates the inflammatory breast cancer phenotype.. Cancer Res.

[9] Clark EA, Golub TR, Lander ES, Hynes RO (2000). Genomic analysis of metastasis reveals an essential role for RhoC.. Nature.

[10] Ikoma T, Takahashi T, Nagano S, Li YM, Ohno Y (2004). A definitive role of RhoC in metastasis of orthotopic lung cancer in mice.. Clin Cancer Res.

[11] Islam M, Lin G, Brenner JC, Pan Q, Merajver SD (2009). RhoC expression and head and neck cancer metastasis.. Mol Cancer Res.

[12] Hakem A, Sanchez-Sweatman O, You-Ten A, Duncan G, Wakeham A (2005). RhoC is dispensable for embryogenesis and tumor initiation but essential for metastasis.. Genes Dev.

[13] Kleer CG, Griffith KA, Sabel MS, Gallagher G, van Golen KL (2005). RhoC-GTPase is a novel tissue biomarker associated with biologically aggressive carcinomas of the breast.. Breast Cancer Res Treat.

[14] van Golen KL, Davies S, Wu ZF, Wang Y, Bucana CD (1999). A novel putative low-affinity insulin-like growth factor-binding protein, LIBC (lost in inflammatory breast cancer), and RhoC GTPase correlate with the inflammatory breast cancer phenotype.. Clin Cancer Res.

[15] Jones RJ, Barber JP, Vala MS, Collector MI, Kaufmann SH (1995). Assessment of aldehyde dehydrogenase in viable cells.. Blood.

[16] Ginestier C, Hur MH, Charafe-Jauffret E, Monville F, Dutcher J (2007). ALDH1 is a marker of normal and malignant human mammary stem cells and a predictor of poor clinical outcome.. Cell Stem Cell.

[17] Charafe-Jauffret E, Ginestier C, Iovino F, Tarpin C, Diebel M (2010). Aldehyde dehydrogenase 1-positive cancer stem cells mediate metastasis and poor clinical outcome in inflammatory breast cancer.. Clin Cancer Res.

[18] Ridley AJ, Hall A (1992). The small GTP-binding protein rho regulates the assembly of focal adhesions and actin stress fibers in response to growth factors.. Cell.

[19] Nobes CD, Hall A (1995). Rho, rac, and cdc42 GTPases regulate the assembly of multimolecular focal complexes associated with actin stress fibers, lamellipodia, and filopodia.. Cell.

[20] Dietrich KA, Schwarz R, Liska M, Grass S, Menke A (2009). Specific induction of migration and invasion of pancreatic carcinoma cells by RhoC, which differs from RhoA in its localisation and activity.. Biol Chem.

[21] Debnath J, Brugge JS (2005). Modelling glandular epithelial cancers in three-dimensional cultures.. Nat Rev Cancer.

[22] Lee GY, Kenny PA, Lee EH, Bissell MJ (2007). Three-dimensional culture models of normal and malignant breast epithelial cells.. Nat Methods.

[23] Kenny PA, Lee GY, Myers CA, Neve RM, Semeiks JR (2007). The morphologies of breast cancer cell lines in three-dimensional assays correlate with their profiles of gene expression.. Mol Oncol.

[24] Reya T, Morrison SJ, Clarke MF, Weissman IL (2001). Stem cells, cancer, and cancer stem cells.. Nature.

[25] Al-Hajj M, Wicha MS, Benito-Hernandez A, Morrison SJ, Clarke MF (2003). Prospective identification of tumorigenic breast cancer cells.. Proc Natl Acad Sci U S A.

[26] Singh SK, Clarke ID, Terasaki M, Bonn VE, Hawkins C (2003). Identification of a cancer stem cell in human brain tumors.. Cancer Res.

[27] Collins AT, Berry PA, Hyde C, Stower MJ, Maitland NJ (2005). Prospective identification of tumorigenic prostate cancer stem cells.. Cancer Res.

[28] Korkaya H, Paulson A, Iovino F, Wicha MS (2008). HER2 regulates the mammary stem/progenitor cell population driving tumorigenesis and invasion.. Oncogene.

[29] Pavlidis N, Pentheroudakis G (2012). Cancer of unknown primary site.. Lancet.

[30] Gupta PB, Onder TT, Jiang G, Tao K, Kuperwasser C (2009). Identification of selective inhibitors of cancer stem cells by high-throughput screening.. Cell.

[31] Abramoff MD, Magelhaes PJ, Ram SJ (2004). Image Processing with ImageJ.. Biophotonics International.

[32] Nocito A, Kononen J, Kallioniemi OP, Sauter G (2001). Tissue microarrays (TMAs) for high-throughput molecular pathology research.. Int J Cancer.

[33] McCabe A, Dolled-Filhart M, Camp RL, Rimm DL (2005). Automated quantitative analysis (AQUA) of in situ protein expression, antibody concentration, and prognosis.. J Natl Cancer Inst.

