# Supplementary material for: RhoC Impacts the Metastatic Potential and Abundance of Breast Cancer Stem Cells
Source: PLoS One. 2012 Jul 23;7(7):e40979. doi: 10.1371/journal.pone.0040979 (PMC3402452; doi:10.1371/journal.pone.0040979)
Supplement: Methods S1 — Supplemental materials and methods. (DOC) [file pone.0040979.s001.doc]

**Supplemental Materials and Methods**

***Reagents***

shRNA was purchased from Open Biosystems through the University of Michigan Life Science Institute High Throughput Screening Core. For western blotting, the following antibodies were used: rabbit anti-RhoC (Cell Signaling), goat anti-actin, and mouse anti-RhoA (Santa Cruz Biotechnology). Matrigel and growth factor-reduced Matrigel were purchased from BD Biosciences. The ALDEFLUOR assay kit was purchased from StemCell Technologies.

***Cell Culture***

Untransfected cell lines were cultured in Ham’s F12 (SUM149) supplemented with 5% fetal bovine serum, or DMEM/F12 (MCF-10A) supplemented with 10% horse serum. Selection media for shRNA-transfected cells consisted of standard cell line media containing 1μg/ml puromycin. Selection media for RhoC or RhoC G14V-transfected cells consisted of standard cell line media with 350μg/ml G418. MCF-10A cells were grown at 37°C in a humidified 5% CO_2_ incubator, and SUM149 cells were grown at 37°C in a humidified 10% CO_2_ incubator.

For 3D cell culture, cells were cultured as described by Lee et al. . Briefly, 4-well chamber slides (Lab-Tek) were coated with growth factor-reduced Matrigel (BD Biosciences). Cells were plated at a density of 2.1 x 10^4^ cells/cm^2^ in cell media containing 4% Matrigel and cultured for 7 days.

**Immunohistochemical Staining and AQUA analysis.**

Briefly, after deparaffinization and rehydration, TMA slides were subjected to microwave epitope retrieval in 10 mM sodium citrate buffer, pH6. After rinsing several times in 10 mM Tris/HCl buffer, pH 8 containing 0.154 M NaCl and 0.05%(v/v) Tween-20 (TBST), endogenous peroxidase activity was blocked with 2.5% (v/v) H_2_O_2_ in methanol for 30 minutes. Non-specific binding of the antibodies was extinguished by a 30 minute incubation with ‘Background Sniper” (BioCare Medical, Concord, CA). The TMA slide was then incubated with a combination of the tumor-specific antibody, cytokeratin (DAKO, Carpinteria, CA, rabbit polyclonal antibody, Z0622, 1:250) and ALDH1 (BD Biosciences, mouse monoclonal antibody, 611195, 1:100) overnight at 4C. The slides were then washed with TBST twice for 5 minutes. The slides were subsequently incubated with an antibody to RhoC (chicken IgY polyclonal antibody, 1:1000) for 60 minutes at room temperature. Slides were then washed as described above and incubated with a combination of goat anti rabbit IgG conjugated to AF555 (Molecular probes, Carpinteria, CA, A21428, 1:200) and goat anti chicken IgY conjugated to AF488 (Molecular probes, Carpinteria, CA, A11039, 1:200) in goat anti mouse Envision+ (DAKO, Carpinteria, CA, K4001) for 60 minutes at room temperature in a dark humidity tray. The slides were then washed as described above and the target image was developed by a CSA reaction of Cy5 labeled tyramide (PerkinElmer, Waltham, MA, 1:50). The slides were washed with 3 changes of water and stained with the DNA staining dye 4’,6-diaminodo-2-phenylindole (DAPI) in a non-fading mounting media (ProLong Gold, Molecular probes, Carpinteria, CA). The slides were allowed to dry overnight in a dark dry chamber and the edges were sealed.

For AQUA, images of each TMA core were captured with an Olympus BX51 microscope at 4 different extinction/emission wavelengths. Within each TMA spot, the area of tumor was distinguished from stromal and necrotic areas by creating a tumor specific mask from the anti-CK protein, which was visualized from Alexafluor 555 signal. The DAPI image was then used to differentiate between the cytoplasmic and nuclear staining within the tumor mask. The pixel intensity of the RhoC protein/antibody complex was determined from the AF488 signal, and finally, the fluorescence pixel intensity of the ALDH1 protein/antibody complex was obtained from the Cy5 signal and reported as pixel intensity.
